# Exposure to parental smoking and child growth and development: a cohort study

**DOI:** 10.1186/1471-2431-13-104

**Published:** 2013-07-10

**Authors:** Seungmi Yang, Adriana Decker, Michael S Kramer

**Affiliations:** 1 The Research Institute of McGill University Health Centre, McGill University, Montreal, Quebec, Canada; 2 Department of Epidemiology, Biostatistics and Occupational Health, McGill University, Montreal, Quebec, Canada

## Abstract

**Background:**

Studies on adverse childhood health and development outcomes associated with parental smoking have shown inconsistent results. Using a cohort of Belarusian children, we examined differences in cognition, behaviors, growth, adiposity, and blood pressure at 6.5 years according to prenatal and postnatal exposure to parental smoking.

**Methods:**

Using cluster-adjusted multivariable regression, effects of exposure to prenatal smoking were examined by comparing (1) children whose mothers smoked during pregnancy with those of mothers who smoked neither during nor after pregnancy and (2) children whose mothers smoked during and after pregnancy with those whose mothers smoked after pregnancy only; effects of postnatal smoking were examined by comparing (1) children whose mothers smoked after pregnancy only with those of mothers who smoked neither during nor after pregnancy and (2) children whose fathers smoked with those whose fathers did not smoke among children of non-smoking mothers after adjusting for a wide range of socioeconomic and family characteristics.

**Results:**

After adjusting for confounders, children exposed vs unexposed to prenatal maternal smoking had no differences in mean IQ, teacher-rated behavioral problems, adiposity, or blood pressure. Children exposed to maternal postnatal smoking had slightly increased behavioral problems [0.9, 95% CI: 0.6, 1.2 for total difficulties], higher body mass index [0.2, 95% CI: 0.1, 0.3], greater total skinfold thickness [0.4, 95% CI: 0.04, 0.71], and higher odds of overweight or obesity [1.4, 95% CI; 1.1, 1.7]. Similar magnitudes of association were observed with postnatal paternal smoking.

**Conclusions:**

No adverse cognitive, behavioral and developmental outcomes were associated with exposure to maternal prenatal smoking. Observed associations with postnatal smoking of both parents may reflect residual confounding by genetic and family environmental factors.

## Background

Exposure to maternal smoking during pregnancy is a well-recognized cause of fetal growth restriction, preterm birth, and oral clefts [1-4]. In childhood, cognitive deficits and problem behaviors such as attention deficit, impulsivity, and conduct problems [5,6] have been associated with maternal smoking during pregnancy; less frequently reported associations include child short stature, obesity, hyperlipidemia, and elevated blood pressure [7-10]. However, studies of these later childhood outcomes have reported far less consistent results, [11,12] although causal relations between exposure to smoking *in utero* and outcomes at birth, particularly for fetal growth, [2] are well established. There also exists a large body of evidence that postnatal exposure to parental smoking is associated with adverse health outcomes in children such as infant mortality, respiratory illnesses, and diet quality [13-15].

Major methodological challenges in determining adverse effects of prenatal and postnatal exposure to parental smoking (particularly for maternal smoking) on offspring health include residual confounding by genetic and environmental factors that are common causes of both parental smoking and offspring outcomes [4,16]. Mothers who smoke during pregnancy have different demographic, psychosocial, and behavioral characteristics from nonsmoking mothers. They tend to be older, are less likely to live with the father of the child, and more likely to be of low socioeconomic position, depressed, and to smoke for a longer period and at higher intensity before pregnancy [17-20]. Moreover, women who smoke during pregnancy tend to continue to smoke afterwards, thus preventing the disentangling of associations with prenatal smoking exposure from those of postnatal smoking. However, few studies have included information on postnatal, as well as prenatal, exposure to maternal smoking [5,21].

Most previous studies have been carried out in industrialized Western countries where the smoking rate has declined or stabilized and is strongly patterned by socioeconomic factors. Examining prenatal smoking effects in a less developed country with different smoking epidemic and different socioeconomic patterning of smoking from Western countries may provide additional evidence bearing on its causal effect on offspring health.

We took advantage of a cohort of early school-age Belarusian children participating in a breastfeeding promotion intervention trial to examine associations between cognitive and behavioral development, growth, adiposity and blood pressure and both prenatal and postnatal exposure to parental smoking. Our extensive data collection includes both prenatal and postnatal maternal smoking and enables us to account for a wide range of potential confounding factors. Moreover, Belarus is a country in economic transition, with low socioeconomic inequality and thus lesser potential for confounding by socioeconomic conditions.

## Methods

### Study participants

Study children were participants in a cluster-randomized breastfeeding promotion intervention trial (PROBIT). A full description of PROBIT’s design and methods has been published elsewhere [22]. In brief, 31 maternity hospitals and their affiliated polyclinics (where children are followed for routine health care) were randomized either to receive a breast feeding promotion intervention modeled on the WHO/UNICEF Baby-Friendly Hospital Initiative or to continue the maternity hospital and polyclinic practices in effect at the time of randomization. A total of 17,046 breast feeding mothers (98-99% of eligible mothers [23]) and their healthy singleton infants born at ≥37 completed weeks of gestation with birthweight ≥2500 g were recruited during their postpartum stay between June 1996 and December 1997. The mother-infant pairs were followed up with research visits at 1, 2, 3, 6, 9, and 12 months; 96.7% (n = 16,491) of them completed the first-year follow-up. A total of 13,889 children (81.5% of the original cohort) were re-examined at 6.5-years with their parents (the mother in 92% of the cases), during which cognitive, behavioral, anthropometric and blood pressure measures were obtained [24-26]. The present study excluded children who did not have cognitive (n = 65), behavioral (n = 82) or anthropometric measures (n = 1 to 17) or who had missing information on at least one confounder (n = 1,513 to 1,911, depending on the outcome), leaving 11,913 to 12,192 children for analysis. The study received approval from the Research Ethics Board of the Montreal Children’s Hospital, and signed consent in Russian was obtained from all participating parents.

### Prenatal and postnatal smoking measures

During enrollment interviews, mothers reported the average number of cigarettes smoked per day during pregnancy (0, 1–4, 5–9, 10–19, ≥20 cigarettes/day). Paternal smoking during pregnancy was not measured. Mothers reported their current smoking status at each visit during the first year with the same categories as in the original questionnaire. At the 6.5-year follow-up, the accompanying parent (usually the mother) reported each parent’s current daily average number of cigarettes smoked (identical categories as for during pregnancy). Given the small number of mothers who reported smoking, maternal smoking was dichotomized as smoking or nonsmoking, both during and after pregnancy. The very small number of maternal smokers and little variations in the number of cigarettes smoked per day prevented us from assessing dose–response relations with the child outcomes. We classified a mother as a postnatal smoker if she reported smoking at any follow-up visit through the 6.5-year follow-up. Based on maternal smoking measures at enrollment and follow-up visits, we cross-classified maternal smoking into 4 categories: smoking neither during nor after pregnancy (“neither”), prenatal smoking only (i.e., smoking during pregnancy but nonsmoking at all follow-up visits), postnatal smoking only (i.e., nonsmoking during pregnancy but smoking at any follow-up visit), and both prenatal and postnatal smoking. Because only 0.5% (n = 72) of the mothers were smoking during pregnancy only and it is unlikely for mothers to smoke during pregnancy only and to quit after birth, we also combined mothers who smoked during pregnancy only with those who smoked during and after pregnancy into the category of mothers smoking during pregnancy. We carried out sensitivity analyses without collapsing the two categories to examine robustness of estimated associations. For paternal smoking, we used both dichotomized categories (smoking vs. nonsmoking) and the full range of categories to examine dose–response relations to outcomes.

### Child outcome measures

At the 6.5-year follow-up, the polyclinic pediatricians administered the Wechsler Abbreviated Scales of Intelligence (WASI) to measure child cognitive ability. [27] The WASI consists of vocabulary and similarities subtests for verbal IQ, and block designs and matrices for performance IQ. Inter-pediatrician agreement was high, with Pearson correlation coefficients of 0.80 (95% confidence interval: 0.67, 0.89) for vocabulary, 0.72 (0.54, 0.83) for similarities, 0.80 (0.67, 0.89) for block designs, and 0.79 (0.66, 0.88) for matrices in a convenience sample of 45 children during a 1-week training workshop provided by child psychologists and psychiatrists [24].

Child behavior was measured using the Strengths and Difficulties Questionnaire (SDQ), [28] a brief behavioral screening questionnaire for children 4 to 16 years that has been validated against other measures of child behavior problems and diagnostic tools of mental disorder in children [29,30]. The SDQ consists of 5 subscales (hyperactivity, conduct problems, emotional symptoms, peer problems, and prosocial behavior), each with 5 items. Each item is rated as not true (0), somewhat true (1), or certainly true (2). Our behavior measures of the SDQ are presented as 4 summarized scores: externalising behaviors (sum of hyperactivity and conduct problem scores), internalising behaviors (sum of emotional symptoms and peer problems), total difficulties (sum of externalizing and internalizing behaviors), and prosocial behavior [31]. Several studies have demonstrated the cross-cultural validity of the SDQ in European and developing countries, [32-35] and the Russian version of the SDQ has previously been used in clinical and research settings [36]. The parental SDQ was completed by the accompanying parent at the 6.5-year follow-up, while the teacher’s SDQ (identical to the parent SDQ) was obtained by mail for children who had begun school by the 6.5-year follow-up. In the present study, we present results of the WASI and the teacher SDQ scores only, because the results of the parent SDQ were very similar to those of the teacher SDQ, and the teacher SDQ may provide a more valid measure of the child’s behavior for this study, since the mother also reported her own and the father’s smoking status.

All anthropometric measurements were obtained in duplicate at the 6.5-year follow-up visit and averaged. [26] These included standing and sitting height, weight, and triceps and subscapular skinfold thicknesses. Body mass index (BMI) was calculated as weight in kilograms divided by height in meters squared; overweight/obesity was defined as age- and sex-specific BMI ≥85th percentile according to 2000 CDC growth charts. Systolic and diastolic blood pressures were also measured in duplicate with a digital oscillometric device (Omron M1) and averaged.

Training and standardization of pediatricians for administering and scoring the WASI and for the anthropometric and blood pressure measurements were assured during the above-mentioned training workshop. The test-retest correlations between measurements at the 6.5-year follow-up visit and re-measurement audits undertaken for 190 randomly selected children after the initial examination were high (Pearson correlation coefficient ≥0.62) for IQ, SDQ, height, body mass index, and waist circumference, especially considering the 18-month average time (range 5.3 to 32.6 months) elapsed between the two measurements, and were modest (0.45 – 0.65) for skinfold thicknesses and blood pressure [24-26].

### Potential confounders

Potentially confounding maternal and family characteristics included maternal and paternal age, marital status, number of older children in the household, maternal alcohol consumption during pregnancy, area of residence (East vs. West Belarus; urban vs. rural), and maternal and paternal education and occupation--all measured at enrollment. We did not adjust for birthweight or neonatal characteristics because they would be intermediate variables rather than confounders. We also included maternal and paternal height (for all outcomes) and body mass index (for anthropometric and blood pressure measures only) based on the parental report at the 6.5-year follow-up. Paternal smoking was included as a confounding variable for analysis of maternal smoking; maternal smoking was accounted for by our restricted analysis of paternal smoking effects to children of never-smoking mothers.

### Statistical analysis

To examine effects of exposure to prenatal maternal smoking, we compared (1) children whose mothers smoked during pregnancy (the combined category of prenatal only and both prenatal and postnatal smoking) to children whose mothers smoked neither during nor after pregnancy and (2) children whose mothers smoked both during and after pregnancy to those whose mothers smoked after pregnancy only. Effects of exposure to postnatal smoking were examined by comparing (1) children whose mothers did not smoke during pregnancy but smoked after pregnancy to children whose mothers smoked at neither period and (2) children of fathers smoked to those of fathers did not smoke at the 6.5-year follow-up only among those whose mother smoked neither during nor after pregnancy.

Because PROBIT is a cluster-randomized trial, we used random-effects linear (for continuous outcomes) and logistic (for overweight/obesity) regression analysis to account for clustering. Associations were sequentially estimated in crude (cluster-adjusted only), partially-adjusted (cluster, sex, and age-adjusted), and fully-adjusted (additionally adjusted for all other confounders) models. We also examined whether the associations differed for boys and girls; no significant interactions were observed (all interactions p-values > 0.1), and therefore we present only sex-adjusted results. We carried out multiple imputation analyses with 5 imputed datasets based on Rubin’s multivariate normal model [37] to assess potential biases due to missing values in our data.

## Results

Table 1 shows the prevalence of PROBIT children’s exposure to prenatal and postnatal parental smoking and their characteristics according to exposure status. As expected, children whose mothers smoked during pregnancy weighed less at birth than those of mothers who did not smoke. In addition, mothers who smoked during pregnancy were more likely to be single at the child’s birth and to have consumed alcohol during pregnancy. Overall, smoking mothers were less educated and more likely to have a manual occupation, and their partners showed similar characteristics. Despite these socioeconomic patterns of smoking similar to those in industrialized Western countries, prevalence of smoking and the extent of inequalities were much smaller in our study (e.g., odds ratio = 1.3 for maternal prenatal smoking among mothers with secondary education or less compared to those with some university education in our data vs. odds ratios = 2 to 4 in other Western countries [18,21]). Mothers were more likely to smoke when their partners were smokers at the 6.5-year follow-up. Mothers who did not attend the 6.5-year follow-up were slightly younger at enrollment (24.0 years vs. 24.4 years), were less likely to have further education after secondary school (62% vs. 64%), and were more likely to have smoked during pregnancy (3.1% vs. 2.1%) than mothers followed up at 6.5 years. In comparison of our analytic sample with those excluded due to missing information on study variables, we observed a lower rate of maternal prenatal smoking (18% vs. 24%). However, there were no differences in other study variables between the analytic sample and those lost to follow-up.

**Table 1:** Prevalence of and characteristics (% of mean (SD)) by pre- and postnatal exposure to maternal smoking among children at 6.5 years of age

	**Maternal smoking**				**Paternal postnatal smoking**	
	**Never smoking**	**Prenatal only**	**Postnatal only**	**Pre- and postnatal**	**Smoking**	**Nonsmoking**
	**(n = 11,927)**	**(n = 72)**	**(n = 1,670)**	**(n = 220)**	**(n = 6,724)**	**(n = 4,295)**
**Prevalence, %**	85.9	0.5	12.0	1.6	61.0	39.0
**Child characteristics**						
Boys	51.7	66.6	50.7	50.9	52.4	51.1
Birthweight, g	3452 (419)	3415 (443)	3377 (421)	3300 (395)	3445 (420)	3471 (418)
Gestational weeks	39.4 (1.0)	39.3 (1.0)	39.3 (1.0)	39.5 (1.1)	39.4 (1.0)	39.4 (1.0)
Age at the follow-up, months	79.3 (3.1)	79.8 (3.2)	79.2 (3.1)	79.8 (3.2)	79.4 (3.3)	79.4 (3.3)
**Family characteristics**						
Maternal age, years	24.7 (4.9)	22.8 (4.5)	23.1 (4.7)	22.4 (4.4)	25.2 (4.9)	25.2 (4.9)
Paternal age, years	27.6 (5.1)	26.4 (5.3)	26.4 (5.0)	25.8 (4.9)	27.3 (5.0)	28.1 (5.1)
Maternal height, cm	164.3 (5.5)	164.6 (5.6)	164.9 (5.7)	164.9 (6.3)	164.6 (5.5)	164.6 (5.5)
Paternal height, cm	176.0 (6.5)	176.0 (6.3)	176.0 (7.0)	176.3 (6.7)	175.7 (6.5)	176.5 (6.5)
Maternal body mass index, kg/m^2^	24.6 (4.3)	24.1 (4.3)	23.5 (4.1)	23.0 (3.6)	24.5 (4.4)	24.5 (4.4)
Paternal body mass index, kg/m^2^	25.7 (3.2)	25.6 (3.2)	25.1 (3.1)	25.5 (4.1)	26.2 (3.3)	26.2 (3.3)
Maternal drinking during pregnancy	1.7	29.1	2.4	19.0	1.9	1.4
Marital status at birth						
Married	91.2	68.1	79.5	55.0	92.4	93.6
Cohabitating	5.8	19.4	12.8	23.6	5.6	4.4
Single	3.0	12.5	7.7	21.4	2.0	2.0
Number of children at home						
0	55.0	80.6	65.0	77.3	54.2	53.8
1	36.0	12.5	28.5	18.6	36.4	37.6
≥2	9.0	6.9	6.5	4.1	9.4	8.5
Maternal education						
University degree	14.3	8.3	6.7	4.1	11.5	19.6
Some university	51.6	52.8	48.0	41.8	50.9	53.3
Secondary	31.0	27.8	38.2	42.7	34.2	25.3
< Secondary	3.1	11.1	7.1	11.4	3.4	1.8
Paternal education						
University degree	13.6	5.1	8.9	2.7	9.7	50.6
Some university	47.4	49.1	48.8	43.8	46.9	42.3
Secondary	36.9	39.0	38.6	43.3	41.0	30.9
< Secondary	2.1	6.8	3.7	10.2	2.4	1.2
Maternal occupation						
Non-manual	45.8	29.2	31.3	17.7	42.4	52.8
Manual	33.9	20.8	33.9	31.8	37.3	27.9
Unemployed	20.3	50.0	34.8	50.5	20.2	19.3
Paternal occupation						
Non-manual	29.0	18.1	23.1	15.0	25.1	36.5
Manual	55.4	43.1	49.0	42.3	60.7	49.0
Unemployed	12.5	19.4	19.6	24.5	12.0	12.1
Don't know	3.1	19.4	8.3	18.2	2.0	2.4
Paternal smoking at the follow-up	61.0	71.2	82.1	78.0		
Residence area, East	47.2	60.7	57.9	56.8	45.8	45.0
Urban	53.6	62.5	73.5	82.3	50.4	56.6

**Figure 1:**
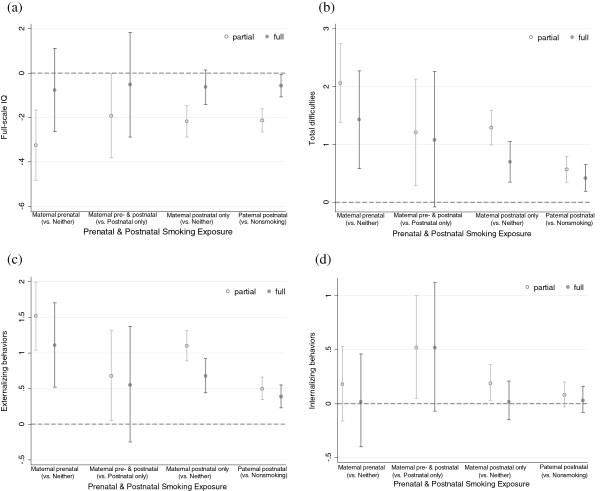
Adjusted* mean differences in child neurobehavioral outcomes at age 6.5 years by exposure to prenatal and postnatal parental smoking. (a) Full-scale IQ. **(b)** Total difficulties. **(c)** Externalizing behaviors. **(d)** Internalizing behaviors. * Partial adjustment model controlled for cluster, child sex and age at outcome measures; full model additionally adjusted for maternal and paternal age, height, education, and occupation, marital status of parents at birth, birth order, maternal drinking during pregnancy, and paternal smoking at 6.5 years age (for maternal smoking only).

Figure 1 presents adjusted mean differences in WASI full-scale IQ (a) and teacher SDQ scores (b – d) according to exposures to prenatal and postnatal smoking in the partly and fully adjusted models. The first two pairs in each graph depict mean differences associated with exposure to maternal prenatal smoking, and the last two, mean differences associated with exposure to postnatal smoking of each parent. For full-scale IQ, exposure to maternal prenatal smoking was not associated with lower IQ scores in both comparisons (prenatal vs. neither prenatal nor postnatal smoking and both pre- and postnatal vs. postnatal only) after adjusting for confounding factors. For SDQ scores, children exposed to maternal smoking during pregnancy had higher total difficulties [1.4, 95% CI: 0.6, 2.3] and externalizing behavior [1.1, 95% CI: 0.5, 1.7] scores compared to those never exposed to maternal smoking, but this association was not observed in the comparison between children exposed to both prenatal and postnatal maternal smoking and those exposed to postnatal maternal smoking only [1.1, 95% CI: -0.1, 2.3 for total difficulties; 0.5, 95% CI: -0.2, 1.4 for externalizing behavior] in the fully adjusted model. Similar results were observed in our sensitivity analyses that we categorized children whose mothers smoked during pregnancy *only* (i.e., exclusion of those whose mothers smoked both during and after pregnancy from the maternal prenatal smoking category); compared with those whose mothers smoked neither during nor after pregnancy, those children whose mothers smoked during pregnancy only had nonsignificantly lower full-scale IQ (−0.2, 95% CI: -3.7, 3.4) and greater behavior problems (0.7, 95% CI: -0.6, 2.0 for total difficulties) in the fully-adjusted models.

For associations of exposure to postnatal smoking, children exposed to maternal postnatal smoking only had slightly lower full-scale IQ scores [−0.6, 95% CI: -1.4, 0.1] and increased total difficulties [0.7, 95% CI: 0.3, 1.0] and externalizing behavior [0.7, 95% CI: 0.4, 0.9] scores compared to those of mothers who did not smoke in both periods after fully adjusting for confounders. Similar associations were observed with postnatal exposure to fathers’ postnatal smoking. Among children of nonsmoking mothers, those exposed to paternal smoking had slightly lower full-scale IQ scores [−0.6, 95% CI: -1.1, -0.1] and elevated total difficulties [0.4, 95% CI: 0.2, 0.6] and externalizing behavior scores [0.4, 95% CI: 0.2, 0.5] after adjusting for confounders than children whose fathers did not smoke. Paternal smoking did not show dose–response relations with the outcomes (data not shown).

Multiple imputation analyses yielded similar results to those results of the complete case analyses above. IQ scores were associated neither with maternal prenatal smoking in both comparisons [e.g., -0.9, 95% CI: -2.4, 0.6 and −0.6, 95% CI: -2.5, 1.2 for full-scale IQ in comparisons 1 and 2, respectively) nor with maternal postnatal smoking [−0.6, 95% CI: -1.3, 0.1], but they were weakly associated with paternal postnatal smoking [−0.5, 95% CI: -1.1, -0.1]. For behavioral outcomes, children exposed to maternal prenatal smoking had higher scores in total difficulties [1.3, 95% CI: 0.7, 2.1] and externalizing behaviors [1.0, 95% CI: 0.5, 1.5] compared to those exposed to neither prenatal nor postnatal smoking of mothers. Both maternal and paternal postnatal smoking exposures were associated with higher total difficulties [0.8, 95% CI: 0.5, 1.1 and 0.4, 95% CI: 0.1, 0.6 for maternal and paternal smoking, respectively] and externalizing behaviors [0.7, 95% CI: 0.5, 1.0 and 0.3, 95% CI: 0.2, 0.5 for maternal and paternal smoking, respectively].

**Figure 2:**
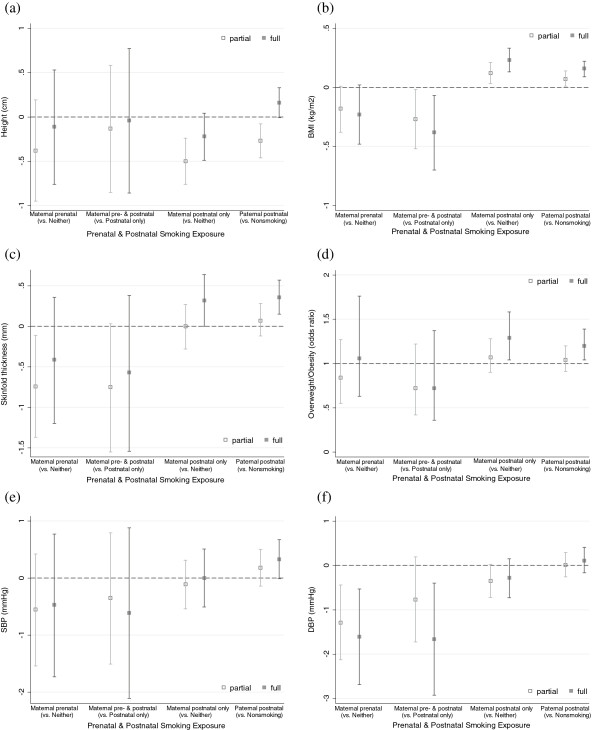
Adjusted* associations of exposure to prenatal and postnatal parental smoking with child anthropometric measures and blood pressure at age 6.5 years. (a) Height. **(b)** BMI. **(c)** Total (triceps + subscapular) skinfold thickness. **(d)** Overweight/obesity. **(e)** Systolic blood pressure. **(f)** Diastolic blood pressure. * Partial adjustment model controlled for cluster, child sex and age at outcome measures; full model additionally adjusted for maternal and paternal age, height, BMI, education, and occupation, marital status of parents at birth, birth order, maternal drinking during pregnancy, and paternal smoking at 6.5 years age (for maternal smoking only).

Figure 2 shows the corresponding associations with selected anthropometric and blood pressure measures (results for other measures are available on request). Overall, exposure to maternal prenatal smoking was not associated with height (a), adiposity (b – d), or blood pressure (e – f) at age 6.5 years. Surprisingly, children exposed to maternal prenatal smoking had slightly lower BMI [−0.2, 95% CI: -0.5, 0.02] and lower diastolic blood pressure [−1.6, 95% CI: -2.7, -0.5] compared to those of neither prenatal nor postnatal smoking mothers after fully adjusting for confounders. The corresponding figures for children exposed to both prenatal and postnatal maternal smoking compared to those exposed to postnatal maternal smoking only were −0.4 [95% CI: -0.7, -0.1] for BMI and −1.7 [95% CI: -2.9, -0.4] for diastolic blood pressure. However, these patterns were not observed for skinfold thickness or systolic blood pressure. When we compared children whose mothers smoked during pregnancy *only* with those whose mother smoked neither during nor after pregnancy, we observed very similar results with wider confidence intervals owing to the small number (data not shown).

Exposure to postnatal smoking was weakly associated with higher BMI [0.2, 95% CI: 0.1, 0.3 for both maternal and paternal smoking], greater total skinfold thickness [0.3, 95% CI: 0.0, 0.6 for maternal smoking; 0.4, 95% CI: 0.1, 0.6 for paternal smoking], and greater odds of overweight/obesity [1.3, 95% CI; 1.04, 1.6 for maternal smoking; 1.2, 95% CI: 1.04, 1.4 for paternal smoking] after fully adjusting for confounding factors. Dose–response relations with paternal smoking were not observed (data not shown). Children exposed to postnatal smoking only showed no differences in blood pressure or height compared to those unexposed to parental postnatal smoking.

Multiple imputation analyses yielded similar results. Maternal prenatal smoking was not associated with any growth or anthropometric measures except diastolic blood pressure [−1.2, 95% CI: -2.1, -0.3] compared to non-smoking in both periods. Postnatal smoking was associated with higher BMI [0.2, 95% CI: 0.1, 0.3 for maternal smoking; 0.1, 95% CI: 0.07, 0.2 for paternal smoking], greater skinfold thickness [0.3, 95% CI: 0.04, 0.6 for maternal smoking; 0.3, 95% CI: 0.1, 0.5 for paternal smoking], and increased odds of overweight/obesity [1.2, 95% CI: 1.0, 1.5 for maternal smoking; 1.1, 95% CI: 1.0, 1.3 for paternal smoking].

## Discussion

In this large cohort of school-age Belarusian children, we examined associations between prenatal and postnatal exposure to parental smoking and cognitive and behavioral development, growth, adiposity, and blood pressure after accounting for a wide range of confounding factors. We found little evidence that exposure to maternal smoking during pregnancy was associated with the child outcomes considered. We observed some associations between postnatal exposure to both maternal and paternal smoking and the childhood outcomes, but the magnitude of these associations was very small.

Although we found associations between maternal smoking during pregnancy and some adverse child outcomes, they were not robust across two comparisons made in our study: children of mothers who smoked during pregnancy vs. those of never-smoking mothers and children whose mothers smoked both during and after pregnancy vs. those of mothers who smoked postnatally only. Positive associations are expected for both comparisons if maternal smoking during pregnancy is indeed biologically associated with offspring outcomes. However, the association was observed only in the comparison of maternal prenatal smoking with never smoking, which suggests no causal role of prenatal smoking in adverse child outcomes. The only exceptions were slightly ***lower*** BMI and diastolic blood pressure among children whose mothers smoked during pregnancy, particularly among children exposed to maternal smoking both during and after pregnancy. We reason that these small effects in magnitude, particularly for lower BMI, are probably due to residual confounding because we found no association with skinfold thicknesses.

Although results from previous studies are inconsistent, our results confirm those of studies that rigorously controlled for confounding factors [16,38-42]. In particular, family-based studies that compared siblings born to the same mothers with discrepant smoking status across pregnancies (to minimize residual confounding by unmeasured family factors) have reported no differences in cognitive ability, externalizing behaviors, or overweight/obesity within siblings [38-40]. Other studies based on large cohorts of children have reported that maternal and paternal smoking during pregnancy are associated to a similar degree with offspring cognitive outcomes [41] and blood pressure [42]. Those results further suggest residual confounding by unmeasured family characteristics, because effects of maternal prenatal (i.e., direct) smoking on offspring should be greater than those of paternal prenatal smoking, which are indirect via maternal inhalation of the father’s cigarette smoke.

We observed small but statistically significant differences in offspring cognitive, behavioral, and anthropometric outcomes according to exposure to postnatal smoking. This finding may suggest that postnatal exposure to parental smoking in childhood is more harmful than prenatal exposure for cognitive and physical development [43,44]. However, we argue that it is more likely to reflect residual confounding by unmeasured confounders. First, our study did not include measures linked to genetic factors such as parent’s psychological states and the presence of psychopathology, which are strongly associated with cigarette smoking; [20] this is an important limitation of our study and of most previous studies. Adoption studies have shown that antisocial personality disorder in biological mothers predicts antisocial behavior in children adopted and raised by prosocial parents [45]. Similarly, recent studies comparing behavioral problems between mother-child pairs who were genetically related and those unrelated as a result of in vitro fertilization also reported increased conduct and attention problems associated with prenatal smoking limited to children of the genetically-related mothers [46,47]. Importantly, those studies found lower birthweight in both groups of children, i.e., those genetically related and unrelated to the mother, thus supporting a causal biological effect of smoking exposure on fetal growth. In addition to genetic transmission of risk, mothers with conduct disorder and/or antisocial personality traits are more likely to be depressed and to provide inadequate parenting and poor-quality interactions, which could result in adverse developmental and health outcomes in children [48].

Second, the lack of a dose–response relation with postnatal paternal smoking also suggests that observed differences in outcome in our study are not caused by cigarette smoke, but that parental postnatal smoking may be a proxy for unmeasured family characteristics. Third, the associations were of similar magnitude with maternal and paternal postnatal smoking. In general, mothers spend more time with their children than fathers; thus the magnitudes of association with maternal smoking, if causal, should be larger than with paternal smoking. This is especially true in our study, because of the standard 3-year maternity leave in Belarus. Even assuming that all fathers who smoked at the 6.5-year follow-up had smoked during the child’s pregnancy and that the effect of paternal smoking was through environmental tobacco smoke inhaled by the mother during pregnancy, the effect of paternal smoking should still be lower than for maternal (active) smoking during pregnancy. However, the associations with paternal smoking were larger than those with maternal smoking during pregnancy and were similar in magnitude to those of postnatal maternal smoking.

Several limitations of our study need to be noted. Parental smoking in our study was primarily based on maternal report of her own and her partner’s smoking. Previous studies comparing biochemical measures and self-reported smoking have largely validated the self-reports, both in general populations and among pregnant women [49,50]. In addition, previous studies have demonstrated good agreement between maternal report of partners’ smoking and data reported by the partners themselves [42]. Nevertheless, misclassification of parental smoking cannot be ruled out as other studies have observed underreporting of smoking among pregnant women [51]. Although no data are available on prevalence of maternal smoking during pregnancy in Belarus, the 2000 Belarusian National Household Survey reported a current smoking prevalence among women of 9%, [52] much lower than that in Western developed countries. A recent meta-analysis of published studies throughout the world estimated smoking cessation rates during pregnancy of 23-43% [53]. Even after taking these data into consideration, prenatal smoking rate in our study is much lower than that of the expected level in Belarus according to the NHS, suggesting under-reporting of smoking during pregnancy in our study. Additionally, there would be potential recall bias of maternal smoking during pregnancy. However, the extent of recall bias would be minimal and non-differential because it was measured during the postpartum stay, not at the 6.5-year follow-up when the child outcomes were assessed. Finally, the lack of associations or negligible associations of prenatal and postnatal exposure to parental smoking we observed may not be applicable to other child health outcomes. Exposure to prenatal and postnatal smoking is clearly harmful for numerous health outcomes in fetuses and children that we did not examine, and pregnant women should still be recommended not to smoke during and after pregnancy.

In conclusion, in this large cohort of healthy Belarusian children born in the mid 1990s, maternal smoking during pregnancy was not significantly associated with offspring cognitive, behavioral, or adiposity outcomes at age 6.5 years. Although exposure to postnatal maternal and paternal smoking was associated with adverse outcomes, those associations were very small in magnitude. Our study provides further evidence that modest associations with childhood developmental outcomes of maternal prenatal and postnatal smoking reflect unmeasured genetic or environmental family factors, rather than biological effects of tobacco exposure, at least for those outcomes considered in our study.

## Competing interests

The authors declare that they have no competing interests.

## Authors’ contributions

Dr. SY conceptualized and designed the study, carried out the analyses, drafted the manuscript, reviewed and revised the manuscript, and approved the final manuscript as submitted. Dr. AD conceptualized and designed the study, carried out the initial analyses, and reviewed and approved the final manuscript as submitted. Dr. MSK conceptualized and designed the study, critically reviewed the manuscript, and approved the final manuscript as submitted. All authors read and approved the final manuscript.

## Pre-publication history

The pre-publication history for this paper can be accessed here:

http://www.biomedcentral.com/1471-2431/13/104/prepub
